# Aroma Formation, Release, and Perception in Aquatic Products Processing: A Review

**DOI:** 10.3390/foods14152651

**Published:** 2025-07-29

**Authors:** Weiwei Fan, Xiaoying Che, Pei Ma, Ming Chen, Xuhui Huang

**Affiliations:** 1 School of Biological Engineering, Dalian Polytechnic University, Dalian 116034, Chinachenming@dlpu.edu.cn (M.C.); 2 SKL of Marine Food Processing & Safety Control, National Engineering Research Center of Seafood, Collaborative Innovation Center of Seafood Deep Processing, Dalian Technology Innovation Center for Chinese Prepared Food, School of Food Science and Technology, Dalian Polytechnic University, Dalian 116034, China

**Keywords:** aquatic products, aroma formation, odorants release, aroma perception

## Abstract

Flavor, as one of the primary factors that attracts consumers, has always been a crucial indicator for evaluating the quality of food. From processing to final consumption, the conditions that affect consumers’ perception of the aroma of aquatic products can be divided into three stages: aroma formation, release, and signal transmission. Currently, there are few reviews on the formation, release, and perception of aroma in aquatic products, which has affected the product development of aquatic products. This review summarizes aroma formation pathways, the effects of processing methods, characteristic volatile compounds, various identification techniques, aroma-release influencing factors, and the aroma perception mechanisms of aquatic products. The Maillard reaction and lipid oxidation are the main pathways for the formation of aromas in aquatic products. The extraction, identification, and quantitative analysis of volatile compounds reveal the odor changes in aquatic products. The composition of aquatic products and oral processing mainly influence the release of odorants. The characteristic odorants perceived from the nasal cavity should be given more attention. Moreover, the relationship between various olfactory receptors (ORs) and the composition of multiple aromatic compounds remains to be understood. It is necessary to clarify the relationship between nasal cavity metabolism and odor perception, reveal the binding and activation mode of ORs and odor molecules, and establish an accurate aroma prediction model.

## 1. Introduction

The rapid growth of the global population and rising incomes have led to a continuous increase in the demand for food. With the gradual scarcity of terrestrial resources, aquatic products are widely recognized in global food systems [1]. Global annual sales of aquatic products exceed $200 billion [2]. Aquatic products have gradually replaced some animal proteins, such as pork and beef, due to their high protein content. Currently, aquatic protein accounts for approximately 7.1–22.3% of all animal protein consumed [3]. Therefore, the development and deep processing of aquatic products with high nutritional value and abundant resources have received increasing attention.

The variability of raw materials and different processing technologies contribute to the specific flavor of aquatic products. It is noteworthy that flavor is of great significance in the processing and consumption of aquatic products. The flavor of aquatic products is composed of odor and taste. Odor is formed by volatile compounds, which can result in olfactory responses. Taste is a gustatory response that can be caused by non-volatile compounds [4,5]. Non-enzymatic browning, fermentation, lipid oxidation, and other cross-linking reactions among the components of raw materials are the main pathways for the formation of specific flavors during the processing of aquatic products [6,7].

The release of odorants aquatic products occurs during their processing, storage, preparation, or consumption. This release typically refers to the release of flavor compounds produced through oral processing [8]. Chemical interactions between components and consumers’ oral processing, such as chewing speed, saliva content, and airflow rate, all affect the odorant release of aquatic products [9]. The matrix of aquatic products can interact with volatile compounds, thus affecting the diffusion of volatile compounds, ultimately reducing their contact with the olfactory organs and altering aroma perception [4]. Moreover, chemical interactions, such as hydrogen bonds, hydrophobicity, ionic or covalent bonds, as well as physical effects, such as the embedding of macromolecular substances and the adsorption of viscous substances in aquatic products, all alter the aroma perception of aquatic products [10].

Aroma perception in the brain is directly related to the ORs of the human olfactory cells [11]. There are approximately 400 known ORs involved in olfactory perception in the human body. All these are G protein-coupled receptors, a type of transmembrane surface receptor [12]. In recent years, many studies have shown that the binding of ORs to odor molecules is non-specific [13]. Aroma perception is not a neural transmission activated by a single OR, but rather a coordinated perception transmission of various receptors [14]. The relationship between various receptors and the composition of multiple aromatic compounds remains to be understood; the relationship between ORs and aromatic compounds remains to be simplified.

This review summarizes aroma formation, release, and perception in aquatic product processing. This work provides meaningful evaluations and conclusions for achieving targeted regulation of the aroma of aquatic products, which will promote the development of the aquatic product processing industry.

## 2. Aroma Formation in Aquatic Product Processing

### 2.1. Aroma Formation Pathways

The Maillard reaction is one of the main pathways for the production of characteristic flavor in aquatic product processing [15]. It belongs to non-enzymatic browning and plays a key role in the formation of aroma during the thermal processing of aquatic products. Numerous studies have been conducted on the products of the Maillard reaction [15,16]. More than 3500 volatile compounds have been identified at different stages of the Maillard reaction, which can form preferred aromas. The main substrates of the Maillard reaction are amino acids and reducing sugars, both of which form imines during heating [15]. These imines further rearrange to form unstable aldose or ketose precursors. These precursors can form heterocyclic aromatic compounds through a series of reactions. Moreover, the precursors of aldehydes or keto sugars may also generate different fragments through aldol reverse condensation, α-cleavage, or β-cleavage. These fragments are prone to condensing with hydroxyl aldehydes or reacting with amino acids to form various heterocyclic aromatic compounds. In addition, Strecker degradation occurs during aquatic product processing [17]. The reaction products can interact with the intermediate products of the Maillard reaction to continuously promote the production of aldehydes, thereby forming a unique aroma. The Maillard reaction plays a crucial role in the formation of characteristic aromas during the thermal processing of aquatic products. In recent years, a large number of scientific researchers have explored the influence of the Maillard reaction on the formation of characteristic aromas during the thermal processing of oysters, mussels, abalones, and squids [18,19,20]. The Maillard reaction is an important means to reduce the fishy smell and other off-odors of aquatic products.

Most aquatic products are rich in lipids; the reaction products of lipids during processing are the key compounds that form the characteristic flavors of aquatic products. The secondary products of lipid auto-oxidation, especially aldehydes, have an impact on flavor [21]. Moreover, lipid degradation products are also important compounds in the formation of the flavor of aquatic products [22]. Furthermore, lipids also undergo β-oxidation, decarboxylation, and cyclization reactions during aquatic product processing, resulting in the conversion of lipids into important flavor compounds such as aromatic ketones and fatty acid lactones [23]. Many studies have shown that lipids and their derivatives play an extremely important role in the generation of the characteristic flavors of aquatic products. For instance, Wang et al. elaborated in detail on the role of lipids in the flavor of scallops [24]. Qiu et al. also found that the key aroma compounds of semi-dried golden pompano, such as 1-octen-3-ol, acetoin and hexanal, were derived from the lipid oxidation and degradation of C18:1, C18:2, C20:2n-6, and C20:4 [25]. However, rancidity and flavor deterioration can be caused by excessive lipid oxidation, which reduces the acceptability of aquatic products. It has been found that changing packaging methods and materials is an effective way to regulate the flavor deterioration under lipid oxidation during the transportation and storage of aquatic products. Rosemary extract has been used as an antioxidant packaging material; such materials have great application potential in controlling the generation of unpleasant odors during the storage of aquatic products [26].

A large amount of lipoxygenase (LOX) is produced during the processing of aquatic products. LOX catalyzes the oxidation of unsaturated fatty acids to form free radicals, which further react with other macromolecular substances to generate key flavor compounds [22]. The polyunsaturated fatty acids in fresh fish meat will form biologically active hydroxyl compounds, such as prostaglandins, under the action of LOX. These hydroxyl compounds further decompose to form the key aroma compounds of fish products, giving them an aroma similar to that of plants.

### 2.2. Effects of Processing Methods on the Aroma of Aquatic Products

#### 2.2.1. Effects of Heating on the Aroma of Aquatic Products

**Figure 1:**
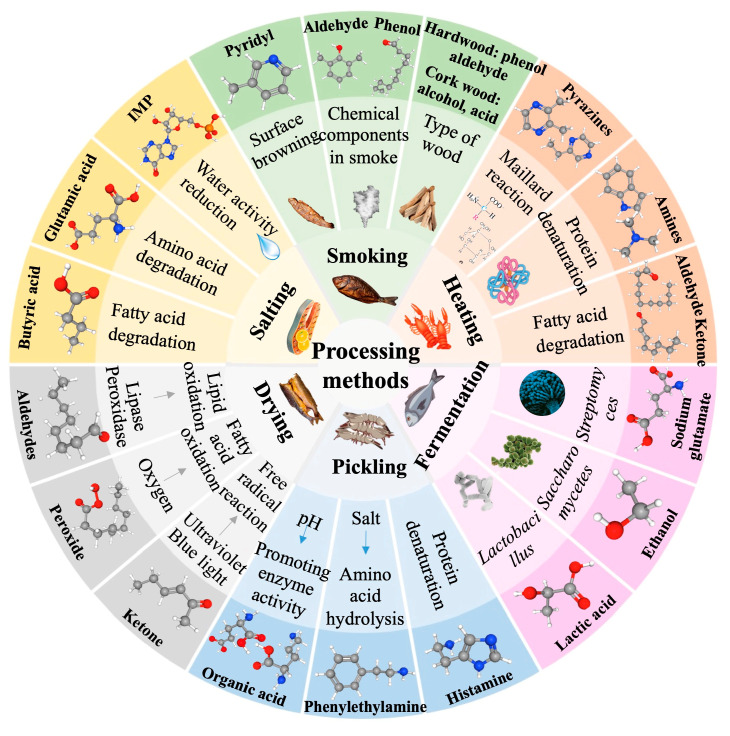
Effects of processing methods on the aroma of aquatic products.

Many low molecular-weight alcohols, aldehydes, and Maillard reaction products are produced during the thermal processing of aquatic products, which together form the unique aroma of cooked aquatic products (Figure 1) [27]. Carbonyl compounds and amines in the aquatic products react with each other. For instance, tuna contains a large amount of ribose, which reacts with cysteine to form 2-methyl-3-furanethiol with heat treatment, resulting in the strong fish flavor in canned tuna [28]. The flavor compounds formed by thermal processing are closely related to the reaction precursors in aquatic products. Lipids in aquatic products may react with carbonyl compounds to form low molecular-weight aldehydes when amines are absent. Key characteristic flavor compounds, such as *N*, *N*-dimethylformamide and *N*-methyl pyrrole, are produced when amines are present. Compounds, such as 2-phenethyl alcohol, 1-penton-3-ol, and dimethyl sulfide, may also be derived from this reaction process; these compounds are also the key aroma compounds in grilled aquatic products [29].

#### 2.2.2. Effects of Salting and Drying on the Aroma of Aquatic Products

In the process of salting and drying, some volatile compounds gradually disperse into the air as the moisture content of the aquatic products decreases; the lipids in aquatic products undergo strong oxidation reactions to generate new aromatic compounds, thus significantly altering the flavor of aquatic products. The flavor of salted and dried aquatic products was significantly correlated with the content of volatile fatty acids [30]. Volatile carbonyl compounds were shown to be the most important flavor components of aquatic products after salting and drying [31]. Previous studies have shown that 142 key volatile compounds have been identified in salted and dried fish, among which carbonyl compounds contribute the most to its flavor [32]. Dried fish is often used as a flavor enhancer in Japanese cuisine. Relevant studies have shown that Japanese dried fish contain 237 key volatile compounds, which contribute to the excellent flavor [33]. These flavor compounds lead to a better sensory experience for consumers.

#### 2.2.3. Effects of Smoking on the Aroma of Aquatic Products

Smoking is a common processing method for most aquatic products, which can effectively improve their flavor. The smoke contains a significant amount of aromatic compounds such as phenols. These compounds gradually adhere and penetrate into the aquatic products’ meat and react with other components, resulting in the characteristic flavor of smoked aquatic products. Sixteen phenolic compounds in smoked salmon have been found, among which guaiacol, 4-ethyl guaiacol, 2,6-dimethoxy phenol, 4-methyl-2,6-dimethoxybenzene, 4-methyl guaiacol, etc., are key flavor compounds [34]. Additionally, 2,4-decadienal, p-xylene, nonanal, and 1-octen-3-ol are the key flavor compounds of Shanghai smoked fish [35].

#### 2.2.4. Effects of Pickling on the Aroma of Aquatic Products

Pickling is also a common processing method for aquatic products, including brine soaking and acidification. Short-chain alcohols, aldehydes, and ketones are the main compounds of the fishy flavor in aquatic products. These compounds can be extracted through the pickling process, significantly reducing the fishy flavor of pickled aquatic products [8]. Relevant studies have shown that, in addition to the short-chain alcohols, ketones, and aldehydes extracted from pickled aquatic products, there are also small amounts of these compounds remaining that play an important role in the flavor of aquatic products [36].

#### 2.2.5. Effects of Fermentation on the Aroma of Aquatic Products

Microorganisms, enzymes, and lipid oxidation are key factors in the formation of the characteristic flavor of fermented aquatic products [21]. The cheese flavor of fermented aquatic products is related to the fatty acids content, while the ammonia-containing flavor is related to ammonia and amine compounds [37]. In addition, the aroma of fermented aquatic products changes with the fermentation broth [38]. Endolipids, alcohols, heterocyclic compounds, and benzaldehyde are the key compounds that form the flavor of fermented aquatic products [4]. Sourness is the characteristic flavor of fermented aquatic products. Acetic acid and *n*-butyric acid are important compounds that form sourness [39]. Different fermentation processes lead to differences in the composition of volatile compounds in aquatic products. In an aroma study of fermented anchovies, the enzymatic reactions of the fermentation process were shown to promote the production of short-chain alcohols, ketones, and aldehydes with green plant and cucumber flavors, while the auto-oxidation reaction leads to conjugated aldehydes with the aroma of oils or greasy or fried-like flavors [40]. Among these, 98 compounds, such as 1-octen-3-one, (E, Z)-2,6-nonadienal, and ethyl 2-methylbutyrate, are the key flavor compounds that constitute fermented anchovies [40].

### 2.3. Characteristic Volatile Compounds in Aquatic Products

**Figure 2:**
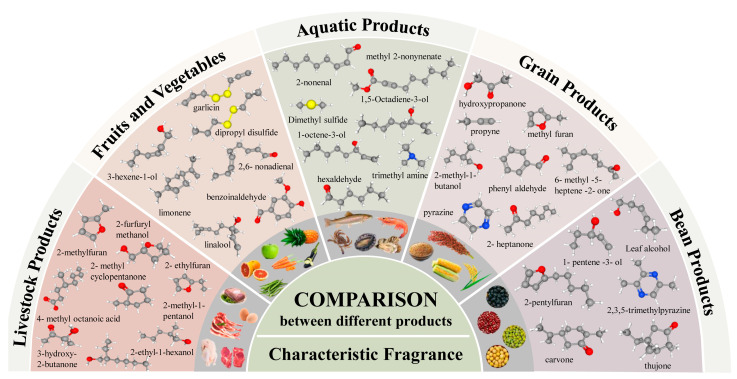
Comparison of the characteristic fragrances of aquatic products to other foodstuffs.

Compared with the fragrances of other foodstuffs, the characteristic fragrances of aquatic products are mainly seafood, fresh, and fishy smells (Figure 2). The unique aroma is mainly derived from rich volatile compounds, including carbonyl compounds, alcohols, nitrogen compounds, sulfur compounds, and hydrocarbons, etc. To date, many volatile compounds with characteristic flavors have been identified in aquatic products. Information on some of the volatile compounds is shown in Table 1.

#### 2.3.1. Carbonyl Compounds

Aquatic products contain a high content of lipase, which can oxidize polyunsaturated fatty acids in aquatic products and produce many carbonyl compounds such as aldehydes and ketones. These compounds together form the characteristic flavor of aquatic products [26]. Aldehydes can reduce unpleasant odors and act as flavor enhancers to increase the flavor intensity of other compounds. (E)-2-nonadienal and (E, Z)-2,6-nonadienal give aquatic products a light cucumber flavor. Ketones usually have a floral and fruity odor, giving the aquatic products an original plant flavor.

#### 2.3.2. Alcohols

The alcohols in aquatic products are mostly derived from the oxidation of fatty acids and have an herbal flavor [23]. The screening and identification of alcohol-characteristic flavor compounds in fresh aquatic products revealed that alcohols, such as 2-octanol, 1-octene-3-ol, and 1-nonene-3-ol, are key compounds [43]. In addition, the content of 1-nonene-3-ol in freshwater fish is lower than that in marine fish, which may be one of the key compounds causing the difference in aroma between marine fish and freshwater fish [44].

#### 2.3.3. Nitrogen Compounds

Pyrazines, pyrroles, pyridines, and trimethylamines are common nitrogen compounds in aquatic products. Trimethylamine is one of the main compounds that make up the fishy smell of aquatic products. The proteins in aquatic products will undergo oxidation and degradation reactions as the storage time extends, thereby generating compounds such as trimethylamine [45]. Pyridine aromatic compounds in aquatic products are usually produced by the dehydration cyclization reaction of amines and aldehydes [46]. The content and odor threshold of pyridine aromatic compounds are both relatively low; thus, these also make an important contribution to the flavor of aquatic products. Pyrazines usually have a roasted dried fruit flavor and are produced during thermal processing, making an important contribution to the flavor of grilled aquatic products.

#### 2.3.4. Sulfur Compounds

Sulfur compounds play a significant role in the flavor of cooked aquatic products. They are generally produced by the chemical reaction between hydrogen sulfide formed by the degradation of sulfur-containing amino acids and other substances in aquatic products. The odor threshold of sulfur compounds is relatively low; their concentrations will directly affect the perception of the final flavor. Dimethyl sulfide makes a significant contribution to the flavor of cooked silver carp, producing a crab meat aroma. However, high concentrations can lead to an unpleasant pungent smell [47].

#### 2.3.5. Hydrocarbons

There are many kinds of hydrocarbon compounds; these are also common in aquatic products. Except for some aromatic hydrocarbons, most hydrocarbons do not significantly contribute to the flavor of aquatic products. Large amounts of saturated and unsaturated alkanes, ranging from C_8_ to C_19_, have been detected in most aquatic products. These alkanes, especially straight-chain alkanes, mostly from the lipids in aquatic products, do not have a characteristic aroma [48].

## 3. Identification Techniques for Volatile Compounds

**Figure 3:**
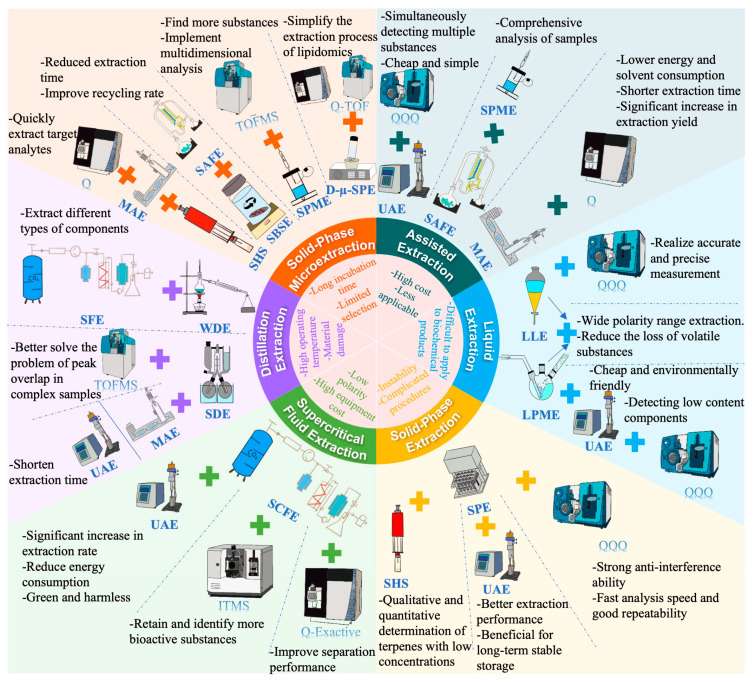
Differences in the identification techniques for volatile compounds.

The flavor of aquatic products is mainly influenced by their key volatile compounds; therefore, it is crucial to obtain the key volatile compounds that constitute the aroma. As shown in Figure 3, the identification of volatile compounds in aquatic products consists mainly of extraction, separation, and identification. Meanwhile, to prove the accuracy and reliability of the identification results, it is necessary to reconstruct the components and verify the accuracy of the results through sensory comparison.

### 3.1. Extraction of Volatile Compounds

The low content and variety of characteristic flavor compounds in aquatic products make accurate analysis relatively difficult [49]. Depending on the volatility and polarity of different aroma components, non-volatile adsorption materials or different types of solvents can be used for extraction, separation, and purification. Currently, the extraction of volatile compounds from aquatic products mainly consists of solvent extraction and headspace extraction. Most of these methods rely on the relative polarity of the aroma compounds; to date, there is no effective method to extract all the compounds. Only the use of several complementary techniques based on different separation capabilities can obtain more comprehensive volatile information.

#### 3.1.1. Solvent Extraction

The release of volatile compounds from aquatic products into the air requires a decrease in solubility and binding or the presence of supersaturation. Therefore, based on the polarity differences, extraction is carried out by changing the dispersion coefficients of volatile compounds between the food matrix and the solvent through different solvents. Commonly used methods include direct solvent extraction, liquid–liquid microextraction, and supercritical extraction. Direct solvent extraction requires further purification of the extract to remove non-scented components, such as water, fat, and protein rich in aquatic products, so as to better separate the volatile substances from the non-volatile residues, which can be achieved by atmospheric distillation or high-vacuum distillation [50]. To simplify and improve this extraction process, simultaneous distillation extraction (SDE), combining solvent extraction and distillation, has been widely used for the extraction and purification of volatile compounds. Although the SDE method effectively extracts some flavor information, it also has many disadvantages such as a low recovery rate, the poor extraction effect of high boiling point substances, and the significant loss of low boiling-point substances. In addition, aquatic products are rich in polyunsaturated fatty acids, which are highly prone to oxidation when heated and produce new aroma substances, thereby leading to false positive results. Solvent-assisted flavor evaporation (SAFE) is a high-vacuum and ultra-low temperature liquid nitrogen freezing process that obtains high boiling-point characteristic aromatic substances by reducing the boiling point of compounds, which is suitable for the characteristic aroma compound extraction of aquatic products. The extracted solvents and volatile compounds remain frozen at ultra-low temperatures, reducing the natural loss of aromatic substances during the extraction process. Therefore, SAFE is also widely used for the extraction and detection of characteristic aromatic compounds in aquatic products. SAFE has shown excellent extraction efficiency in aroma studies of fish products [51].

#### 3.1.2. Headspace Extraction

Volatility is an inherent property of aromatic compounds. Within a certain range of space, temperature, and pressure, the distribution coefficients of aroma substances in the food matrix and food top space will reach a certain balance. The aroma substances of the top space were analyzed by using this balance to obtain part of the characteristic aroma substance information. Headspace extraction eliminates the impurities introduced during solvent extraction and the chemical reactions between odorants and solvents [52]. The information on volatile compounds obtained is closer to the aroma composition directly emitted from food. Although the advantages of headspace extraction are obvious, due to problems such as the low headspace release of aroma substances caused by the high water content in aquatic products, the number of accurate aroma compounds that can be obtained by headspace extraction is fewer than those obtained by solvent extraction. As a result, the information on volatile compounds obtained by headspace extraction is not comprehensive enough, which also makes it difficult to extract and identify many key aroma compounds with low contents.

According to the differences in headspace extraction methods, headspace extraction can be roughly divided into static headspace (SHS) and dynamic headspace (DHS). SHS usually uses an extractor with an extraction coating to perform at a certain temperature and the operation is relatively simple. Among these, solid phase microextraction (SPME) utilizes different adsorption coatings to adsorb the headspace aroma of food and has been widely used in the detection and analysis of various food volatile compounds due to its convenience [52]. However, due to the limitations of the adsorption capacity and extraction efficiency of the adsorption coating, although SPME has a good potential for flavor analysis, its extraction effect on some aroma substances is not ideal, especially for meat and aquatic products. The low content of volatile compounds in food headspace is one of the factors affecting the effectiveness of SHS. To reduce the influence and concentrate the obtained headspace volatile compounds, DHS uses inert gas to bring out the characteristic aroma compounds in the aquatic products’ headspace and collects them in a capture well with adsorbent materials to achieve purification, capture, and the detection of other low-content characteristic volatile compounds in aquatic products [52]. DHS is also widely used for the aroma analysis of fish oil due to its superior extraction efficiency [53].

### 3.2. Identification of Volatile Compounds

Gas chromatography–mass spectrometry (GC–MS) has been the optimal method for the analysis of volatile compounds due to its superior separation of gaseous substances and high detection sensitivity [54]. Mass spectrometry has gradually become prominent in food aroma identification applications due to its superior sensitivity and good tandem application with separation chromatography [54]. However, the complexity of volatile compounds determines that the GC–MS method cannot fully satisfy the identification of all compounds. Therefore, many methods have been continuously optimized and improved in chromatography or mass spectrometry, which provide valuable additional or supplementary information for the identification of volatile compounds.

#### 3.2.1. Gas Chromatography–Olfaction (GC–O)

As important as the identification of odorants is to determine whether the compounds have odor or contribute to the overall aroma. Although GC–MS can effectively identify volatile compounds, it cannot determine whether the compounds have aroma contributions [54]. To make up for the deficiency of GC–MS in the identification of odorants, based on the separation of GC and combined with human olfactory perception, the GC–O was formed. In the process of GC–O or GC–MS/O detection, the experimenter can judge the odor perception and describe information such as odor intensity, odor descriptors, etc. Moreover, many methods for determining the aroma of volatile compounds have emerged in food flavor analysis, such as charm value analysis (Charm), time-intensity method (Osme), and aroma extraction dilution analysis (AEDA), which can effectively determine the contribution of volatile compounds to flavor [55]. In addition, based on the accurate identification and quantitative analysis of key compounds, the calculation of odor activity value (OAV) can be used to a certain extent to determine the key molecular factors for the potential aroma generation of a certain type of food.

#### 3.2.2. Comprehensive Two-Dimensional Gas Chromatography

The comprehensive two-dimensional gas chromatography and time-of-flight mass spectrometer (GC×GC–QTOF) is a high-performance instrument for complex sample analysis [56]. The advantages of GC×GC–QTOF are mainly reflected in high separation capacity, high resolution, multi-dimensional analysis, and rapid detection, etc. The GC×GC is composed of polar and non-polar columns, which is mainly addresses the issue of the severe insufficiency of peak capacity in traditional one-dimensional gas chromatography when separating complex samples [57]. It can significantly improve the separation effect of trace substances in complex matrixes. In recent years, GC×GC–QTOF combined with multivariate data analysis has been applied to identify volatiles of different aquatic products, such as grilled eel, hybrid catfish, smoked mackerel, tilapia fillets, etc. [58,59]. It is meaningful and convenient for analyte identification and sample fingerprinting.

#### 3.2.3. Chiral Gas Chromatography

Among the odorants, many compounds have corresponding isomers. Due to the differences in their chiral structures, these corresponding isomers may cause differences in their aroma perception. However, the separation effect of traditional gas chromatography columns for compounds with chiral structures is not ideal; a special chiral gas chromatography column is normally used for separation [60]. Chiral separation is usually performed using chiral gas chromatography columns with cyclodextrins as adsorbent coatings during the analysis. To improve the separation efficiency of compounds, they are typically connected in series with other types of gas chromatography columns to form multidimensional chromatography. Although chiral gas chromatography is widely used in the analysis of complex odorants, its application in the comprehensive analysis of aquatic products is relatively rare.

#### 3.2.4. High-Resolution Mass Spectrometry

The superior sensitivity and accuracy of high-resolution mass spectrometry enable it to accurately obtain the mass number and elemental composition of compound ion fragments during the detection, which is of great significance for the verification of the identification results of volatile compounds. High-resolution mass spectrometry, such as time-of-flight mass spectrometry (TOF), Orbitrap, magnetic mass spectrometry, and Fourier transform–ion cyclotron resonance mass spectrometry (FT-ICR), has been gradually applied to food flavor analysis; a high-resolution mass spectrometry database has also been accumulated [61].

#### 3.2.5. Chemical Ionization (CI) Mass Spectrometry

CI mass spectrometry is usually used as an auxiliary means for the identification of volatile compounds. The CI ion source belongs to the soft ionization ion source. It ionizes chemical reaction gases such as methane and ammonia, causing ionic reactions with the compounds to be measured during the detection process, resulting in additional ion fragments, such as (M + H)^+^, etc. The identification of these fragments can obtain the parent ion information of the compounds to be measured that cannot be obtained by electron impact ionization (EI) ion sources. Therefore, CI sources are often used instead of EI sources to detect the parent ion information of volatile compounds. The identification results can be further verified by comparing the mass numbers of the compounds.

#### 3.2.6. Proton Transfer Reaction–Mass Spectrometry (PTR–MS)

PTR–MS is currently becoming increasingly mature in the field of food analysis. Proton transfer reaction (PTR) is a chemical ionization source technology based on proton transfer. This technology usually uses H_3_O^+^ as the proton donor to transfer protons to trace gas molecules. It has the advantages of high ionization efficiency and less fragmentation. PTR–MS provides a rapid real-time monitoring with high sensitivity at ppt levels. It also introduces a switchable reagent ion function, enabling primary ions to switch from H_3_O^+^ to NO^+^ and O^2+^ within seconds. PTR–MS has advantages in the study of volatile compounds released by various foods such as surimi, grilled fish, smoked fish, etc. [62]. One of its key applications is to analyze food chemical processes over time such as the formation process of aroma in fish during roasting, and the extension and changes of taste and aroma in the nasopharynx during eating. In addition, some long-term studies that require continuous observation, such as the spoilage of fish and the quality loss of food after oxidation, can also be conducted with the help of PTR–MS.

### 3.3. Quantitative Analysis of Volatile Compounds

Different compounds have different odor thresholds. Whether the odor threshold is reached directly affects people’s perception of the aroma. Thus, the calculation of the content of aroma compounds is of great significance for aroma analysis. In the process of determining aroma compounds, it is necessary to select an appropriate quantitative analysis method [63]. This not only requires considering the error of quantitative results but also the cost of quantitative analysis, as well as standards and internal standards. According to the statistical methods of results comparison and quantitative analysis, it can be roughly divided into the normalization method, external standard method, and internal standard method.

#### 3.3.1. Normalization Method

The normalization method is a chromatographic quantitative method that calculates the content of each compound by comparing the chromatographic peak sizes of all the compounds to be tested. When the injection volume is less than the loading range of the chromatographic column, the quantitative results are related to the purity of the sample and the chromatographic separation effect. Due to the differences in the extraction efficiency of volatile compounds, the response speed of the instrument, and the chromatographic separation efficiency in the process of aroma analysis, the quantitative results differ from those of the actual samples. When the correction factor of each compound to be quantified is quite different, the quantitative process needs to consider the influence of different responses of the instrument on the quantitative results. Therefore, it is necessary to introduce a correction factor to the calculation. When the correction factor of the compound to be quantified is relatively close, the influence of the instrument response on the quantitative results can be ignored. The advantages of this method are that it is suitable for small-scale injections and that changes in the instrument and operating conditions have a relatively slight influence on the results.

#### 3.3.2. External Standard Method

The external standard method converts the concentration of a compound by comparing its response intensity with that of a standard of known concentration. Compared to the normalization method, this method does not require the calculation of a correction factor. The accuracy is more dependent on the reproducibility of the experiment and the stability of the instrument. Thus, it is necessary to keep the concentration of the control solution as similar as possible to the compound to be measured when performing the external standard method. Under the same experimental conditions, the concentration of the compound to be measured can be calculated by using the regression equation of the standard curve based on the peak area or peak height of the component to be measured. The advantage of this method is that it is simple to operate and calculate; no internal standards are added.

#### 3.3.3. Internal Standard Method

The internal standard method is a commonly used quantitative approach that uses a substance without the compounds to be measured as an internal standard for error correction during extraction and detection. In the actual sample analysis, the compound is detected under the same conditions as the standard curve to obtain the peak area or peak height of the internal standard and the compounds to be measured, and the corresponding concentration is calculated by the regression equation. The advantages of this method are that the experimental error is minor and the results are highly comparable. Currently, isotopic internal standards are gaining importance in the quantitative analysis of odorants due to their superior stability and similar physicochemical properties to those of the compounds to be measured, especially those labeled with ^13^C or ^2^H [64]. Qian et al. compared the characteristic volatile compounds of different surimi samples by using isotopic internal standards [65]. However, due to the difficulties in the synthesis, isolation, and purification of isotope internal standards, as well as the diversity and complexity of odorants, the cost of substance quantification using all-isotope internal standards is very high. Moreover, it is also difficult to synthesize stable isotope internal standards for some compounds. Therefore, the selection of internal standards should be based on the magnitude and acceptability of quantitative errors, rather than relying solely on stable isotopes as the standard.

## 4. Odorant Release of Aquatic Products

### 4.1. Effects of the Composition of Aquatic Products on Odorants Release

The release of volatile compounds in food is one of the important factors that stimulate the sensory characteristics of aroma. The flavor of the final product is determined by combining the specific aroma of the volatile components in the raw materials. The release of aroma is influenced by the physicochemical properties (hydrophobicity) and thermodynamic factors (such as partition coefficient) of volatile compounds, the interaction between aroma and the food environment (lipids, proteins, sucrose, etc.), as well as the temperature during consumption. These factors jointly affect the perceived intensity of aroma in food.

#### 4.1.1. Effects of Lipids on Volatile Compound Release

Lipids usually do not interact chemically with volatile compounds but merely act as solvents, reducing the rate of the diffusion of volatile compounds in the air over aquatic products through solubility [23]. Liquids in the aqueous phase will dissolve some of their lipophilic compounds, thereby reducing the number of lipophilic compounds in the aqueous phase and weakening their motivation to diffuse outward. Conversely, when predominantly in the lipophilic phase, the presence of the aqueous phase will also have an impact on the diffusion drive of the corresponding substances.

#### 4.1.2. Effects of Carbohydrates on Volatile Compound Release

The influence of carbohydrates on volatile compounds is more complex, encompassing not only the possible chemical interactions with volatile compounds but also the physical rheology, viscosity, and other properties that affect the transport of aroma compounds in aquatic products [66]. The carbohydrate-rich side-chain groups can provide many chemical binding sites for volatile compounds, such as COO^−^, NH_4_^+^, and glycoprotein peptide side chains, and the lipophilic binding sites of octenyl succinate-derived starches [66]. In addition, some carbohydrates may have certain conformational forms that provide hydrophobic binding sites such as the hydrophobic nucleus of cyclodextrin. Thus, the interaction between carbohydrates and aromatic compounds can affect the volatility of the aromatic compounds and reduce their vapor pressure. The magnitude of the interaction and the reduction in the vapor pressure of the volatile compounds mainly depend on the composition of the volatile compounds and the types of carbohydrates.

#### 4.1.3. Effects of Protein on Volatile Compounds Release

Proteins are also rich in side-chain groups, which can chemically interact with volatile compounds, thereby affecting the release of volatile compounds. The physical properties of proteins, such as emulsification and foaming, can also influence the mass transfer effects and change the release rate of volatile compounds [67]. Moreover, protein structures, such as amino acid side chains, protein terminals, and hydrophobic pockets, provide more and more complex sites for reactions with aroma compounds. Furthermore, factors that form physical barriers to the mass transfer of aroma compounds, such as viscosity and gel structure, are usually associated with proteins.

Hydrolyzed proteins have no hydrophobic binding effect and bind less effectively to the aroma compounds than intact proteins. However, the intrinsic chemical reactivity with aroma components has not decreased. A large number of terminal amino groups and new side chains are exposed after protein hydrolysis. Hydrolyzed proteins are more likely to react with carbonyl groups, while disulfide bonds interact more strongly with aroma compounds.

### 4.2. Effects of Oral Processing on Volatile Compound Release

The aroma of food perceived by humans during the eating process is mainly affected by the speed and extent of the release of volatile compounds in the oral cavity, while the physical and chemical reactions that occur during oral processing are direct influencing factors [68]. Moreover, saliva alters the distribution of aroma among food, saliva, and air by protein hydration, dilution, or binding to specific volatile compounds, thus influencing the diffusion of aroma into the nasal cavity [69]. Furthermore, the touch pressure of the tongue on food can influence the release of odorants; this effect is directly related to the magnitude of the touch pressure [70]. Although many studies have attempted to reveal the mechanisms of aroma perception during oral processing, many of these mechanisms remain poorly understood due to individual differences and complex oral environments.

During the process of consuming aquatic products, the perception of aroma by the sense of smell is highly dependent on oral parameters. To monitor the release and perception of odorants in food during consumption, three online analytical instruments, atmospheric pressure chemical ionization–mass spectrometry (APCI–MS), selected ion flow tube–mass spectrometry (SIFT–MS), and PTR–MS, are used for the real-time monitoring of odorants in the low-concentration air of cavities and nasal cavities [71]. However, there are some major limitations in the study of odorants release in vivo such as significant differences within and between individuals. To overcome these limitations, some artificial devices have been developed to imitate humans chewing food such as artificial saliva and chewing simulators. However, the aroma released from food substrates, bolus, or salivary-liquid substrates is dynamic. Developing more accurate and suitable equipment to simulate the process of oral processing is an important research direction for studying the characteristic aroma compounds released during food consumption.

### 4.3. Physicochemical Models of Volatile Compound Release

One of the major challenges that flavorists face is to create the same aroma from liquid foods (such as fish soup), semi-solid foods (such as fish sauce), or solid foods (such as fish meat). Modeling can help to predict the release or retention of aroma compounds in aquatic products. Mathematical models based on physical and chemical properties and molecular conformational models based on atomic group interactions are the main methods for exploring the release of aroma components in aquatic products. To achieve this goal, mathematical models based on physical and chemical properties, such as diffusion, transfer rate, and volatility, were established. One classic equation used is to estimate the volatility of aromatic compounds in an oil/water/air three-phase system, considering the distribution coefficients of oil/air and water/air, as well as the volume fractions of oil and water in the mixture. In the process of simulating the release of aromatic compounds from unstirred aqueous solutions, it was found that the aroma compounds existing in the air above the food were diluted by the gas flow. The release of aromatic compounds depends on the vapor–liquid partition coefficient, the liquid–vapor two-phase mass transfer coefficient, and the gas flow rate. The two-layer theory, a theoretical model for calculating the release of aroma compounds in homogeneous solid foods, can simulate the mass transfer process of food compounds in the oral cavity [72]. This model considers the release of odorants from the solid phase to the aqueous phase. For aquatic products where there is an interaction between aromatic compounds and macromolecules, the release rate mainly depends on the affinity constant. In most cases, the limiting step for aroma release is the mass transfer process at the liquid–gas interface.

## 5. Aroma Perception of Aquatic Products

The ORs of human olfactory cells are directly related to the formation of aroma perception in the brain. Aromatic compounds enter the olfactory organs of the body through the anterior and posterior nasal passages, stimulating the olfactory receptor cells in the nasal cavity to produce specific ORs and bind to them. When the ORs combine with volatile compounds, the olfactory cells produce specific nerve impulses, which are transmitted to the olfactory bulb through nerve conduction and amplified by the olfactory cortex of the brain [73].

### 5.1. Nasal Metabolism and Olfactory Perception

Aroma perception is a complex process. There are differences in the odor perception process of volatile compounds through the anterior nasal cavity and the posterior nasal cavity during consumption. At the same time, when perceiving certain odors for a long time, there may be phenomena such as odor perception fatigue and decreased perception sensitivity. Based on these phenomena, scientists have put forward many conjectures, among which the hypothesis of enzyme metabolism in the nasal cavity has been widely recognized [74]. The nasal mucosa of the nasal cavity contains very active, non-specific enzymes. According to their functions, they can be roughly classified into enzymes that catalyze oxidation or reduction enzymes, catalytic hydrolases, and enzymes that catalyze molecular-binding reactions [74]. Many studies have shown that a series of metabolic reactions occur after odorants enter the nasal cavity, which may be related to olfactory perception; however, there is no direct evidence indicating a relationship between nasal cavity metabolism and odor perception [75]. This is because the complex enzyme system and environment in the nasal mucosa result in a complex metabolic process; it is difficult to establish a direct link between nasal metabolism and transient odor perception.

To explore the relationship between nasal metabolism and olfactory perception, many researchers have proposed corresponding metabolic hypotheses based on the identification of nasal enzyme systems. Moreover, it has been suggested that the production of the characteristic aromas of volatile compounds may be related to the types of metabolites produced in the nasal cavity [76]. Volatile compounds may not directly bind to Ors; however, different types of metabolites may bind to ORs to activate aroma perception. Therefore, the metabolism of odor molecules in the nasal cavity may be related to differences in the human olfactory perception of odorants. Although the olfactory mucosa has a high metabolic activity for odorants, and the corresponding metabolites can be detected in the mucus of the nasal mucosa, there are still many blind spots in the olfactory theory regarding the possible role of these metabolites in improving odor quality, which requires further validation. Hung et al. utilized APCI–MS to explore differences in the perception of the characteristic aroma components of grilled eel through the anterior and posterior nasal cavities during consumption [77]. The study demonstrated the influence of the posterior nasal cavity’s perception of trimethylamine, 1-penten-3-ol, and 2-methyl-1-butanol on the sensory perception of the characteristic aroma of grilled eel.

### 5.2. Research Progress on Aquatic Products Aroma Perception by ORs

ORs, as signal elements on the cell membrane of olfactory sensory nerves, sense odor molecules from the outside of the cell [78]. Current research on ORs has focused on both gene expression and protein structure. More than 2% of the genes encoding proteins in humans are OR genes [79]. However, only 396 functional human OR genes are currently thought to be capable of aroma information transmission. Due to the variability in gene expression, the changes in individual bases lead to differences in the interaction between odor molecules and ORs. Therefore, everyone’s perception of the same odor may be different [80]. The OR genes of mammals are divided into two categories. One category of genes encodes ORs that mainly recognize hydrophilic aromatic compounds, while the other category of genes encodes ORs that mainly recognize hydrophobic aromatic compounds [81].

ORs are located on the membranes of olfactory sensory nerve cells. Studies have shown that each olfactory sensory nerve cell may express only one OR, mainly due to the different chromatin positions of individual alleles of ORs [82]. The expression of the OR genes generates a negative feedback signal, thereby inhibiting the expression of other OR genes and enabling olfactory sensory nerve cells to express a single OR through gene silencing [83]. Moreover, some studies have also found that multiple types of ORs are expressed in single olfactory sensory nerve cells, suggesting that the activation of the ORs gene is complex. It may be a specific single expression or co-expression [84]. The role of ORs in nasal epithelial cells is to detect and distinguish odor molecules based on combined codes. Each OR can detect multiple odor molecules; odor molecules can also activate various ORs. Thus, a mixture of various odor molecules can activate a specific group of ORs and there is partial overlap among the ORs activated by different odor molecules.

ORs normally consist of 320 ± 25 amino acids. Minor modifications or changes in amino acid structures can lead to alterations in the binding of ORs to odor molecules. These changes are among the key determinants of whether the olfactory perception cascade reaction occurs [85]. Therefore, although the binding between ORs and odor molecules is non-specific, the subtle differences in the spatial structure of ORs lead to variations in the binding capacity of each OR to different odor molecules. These differences lead to competition among odor molecules for the activation of ORs. Odor molecules that preferentially bind and activate ORs will generate negative feedback, inhibiting the re-binding of the OR with other odor molecules, thereby further suppressing the generation of certain odor signal perceptions [86]. Although there are a large number of odor compounds in nature, far exceeding the number of ORs, the non-specific binding and competitive activation of ORs result in different odor perceptions when exposed to different odor molecules [87].

In recent years, the rise of artificial intelligence technology (AI), including machine learning and molecular docking, has completely transformed research on olfactory perception [88]. The combination of protein homology modeling and molecular docking has been applied to predict odorant–receptor interactions [88,89]. The binding of ORs to odor molecules is also a ligand-receptor binding model. The relationship between different ORs and volatile compounds can be analyzed by molecular docking techniques [90]. The physicochemical structure of ORs and odorants directly determines the quantity, type, and duration of OR activation during each odor perception. Although there are certain problems with the accuracy of the structural relationship between ORs and odor molecules obtained by using homology modeling and molecular docking techniques, it still has reference value for exploring the binding mode between ORs and odor molecules. In a study on the perception of the characteristic odors in fish meat, Xia et al. utilized the homology modeling and molecular docking techniques of typical OR proteins to explore the influence of the perception differences in key odor compounds in fish meat on the characteristic flavors of fish meat [91].

In recent years, many typical ORs have gradually been applied in the analysis and research of the characteristic odor perception of aquatic products. OR1A1 plays a key role in the detection of various odor compounds [92]. It has a high correlation with the odor perception of the interaction between odor compounds and fish aroma. OR1D2 is also involved in the perception of various volatile compounds in aquatic products, especially aldehydes, which make significant contributions to the characteristic aroma of fish [91]. OR2J3 can detect amines and other nitrogen-containing compounds in fish muscles, which affect their off-odor. OR2W1 has been identified as a broad-spectrum OR that mainly reacts to terpenoids, which may contribute to the natural aroma of fish muscle and enhance our understanding of its sensory perception. In a study on the perception of characteristic aromas after surimi maturation, Zhai et al. conducted molecular docking simulations using Ors, such as OR2W1, OR2T11 and OR2T33, and odor compounds, such as octanal, decanal, 3-methylbutanal, heptanal, (Z)-4-heptanal etc., and analyzed the possible relationship between the content of odor molecules and perceived intensity [93].

## 6. Future Perspectives

Odor is a crucial indicator for evaluating the quality of aquatic products and attracting consumers. For aquatic products, off-odor and aroma have always been two important criteria for judgment. The off-odors represented by fishy odors limit the utilization, development, and consumption of aquatic products and their processed derivatives. Therefore, reducing the intensity of off-odors and enhancing and maintaining characteristic aromas have always been important development directions for aquatic product processing technology. The odor perception of consumers for aquatic products is divided into two steps: one is the sniffing before consumption, and the other is the odor perception during consumption. In recent years, a large number of studies have continuously utilized technologies, such as GC–MS, to explore the characteristic odorants of various aquatic products, seeking the reasons behind the differences in prenasal olfactory perception at the molecular level and formulating characteristic aroma fingerprint profiles for different products, in order to guide the regulation of aroma generation in aquatic products during processing. In addition, with the rise of related technologies, such as PTR–MS, it has become possible to perceive the dynamic aroma of aquatic products during consumption and observe the aroma compounds released during the consumption process, which also provides a theoretical research basis for the sensory improvement of the flavor quality of aquatic products. Based on the identification of off-odor compounds and aroma compounds in aquatic products, the removal of off-odor components, such as fishy components, and the improvement in typical aroma components, like aldehydes, ketones, and alcohols in aquatic products, have been achieved through processes such as pickling, microbial deodorization, controllable Maillard reaction, controllable lipid oxidation, etc. In addition, based on structural reorganization, adjusting the spatial structure relationship between odor substances and components, such as proteins and lipids, is also an important direction for regulating the release and perception of aroma in aquatic products. Furthermore, studying OR activation in vivo, designing delivery systems for aroma stability, and establishing a more accurate aroma prediction model for AI flavoring are also key research directions in the future. Therefore, it is important to understand the formation and control of the odor compounds in aquatic products and to develop targeted adjustments for processing, packaging, and the storage of aquatic products in order to meet the needs of consumers.

## Figures and Tables

**Table 1:** Key flavor compounds in aquatic products [41,42].

Classification	Name	Molecular Formula	Flavor Description	Threshold
Carbonyl Compounds	Butanedioic acid diethyl ester	CH_3_CH_2_OCO(CH_2_)_2_COOCH_2_CH_3_	Characteristic odor	100.0000
	Ethyl Formate	HCOOC_2_H_5_	Fruity, sharp, rum-liker odor	90.9000
	3-Pentanone	CH_3_CH_2_COCH_2_CH_3_	Acetone odor	3.0000
	Heptanal	C_7_H_14_O	Penetrating fruity odor	2.7000
	2-Acetone	C_3_H_6_O	Fragrant, mint-like odor	2.0000
	Methyl palmitate	C_17_H_34_O_2_	Fruit, sweet, slightly fatty odor	2.0000
	2-Methyl-propanoic acid	(CH_3_)_2_CHCOOH	Sharp, butter-fat-like odor	1.5000
	2-Butanone	C_4_H_8_O	Moderately sharp, fragrant, mint or acetone-like odor	1.3000
	Propanoic acid	CH_3_CH_2_COOH	Pungent, disagreeable, rancid odor	1.0000
	Benzaldehyde	C_6_H_5_CHO	Odor resembling oil of bitter almond	0.7510
	Butyl butyrate	C_8_H_16_O_2_	Fruity, pineapple-like odor	0.4000
	Hexanal	C_6_H_12_O	Fatty-green, fruity odor	0.2300
	Octanoic acid-methyl ester	C_9_H_18_O_2_	Powerful, winey, fruity, orange-like odor	0.2000
	3,5-Octadien-2-one	C_8_H_12_O	Pungent herbaceous odor	0.1500
	Butyric acid	CH_3_CH_2_CH_2_COOH	Penetrating and obnoxious odor	0.1450
	(E, E)-2,4-Heptadienal	C_7_H_10_O	Fatty, green odor	0.0570
	2-Octanone	C_8_H_16_O	Fatty, green cheese, fruity odor	0.0502
	2-Nonanone	C_9_H_18_O	Fruity, floral, fatty, herbaceous odor	0.0320
	3-Octanone	C_8_H_16_O	Mild fruity odor	0.0214
	Acetic acid	CH_3_COOH	Sour, vinegar-like odor	0.0130
	Methyl butanoate	C_5_H_10_O_2_	Apple-like odor	0.0059
	2-Undecanone	C_11_H_22_O	Citrus, fatty, rue-like odor	0.0055
	1-Hexanone	C_6_H_12_O	Sharp, fruity, green grass odor	0.0050
	Decanoic acid ethyl ester	C_12_H_24_O_2_	Oily brandy-like odor	0.0050
	Decanal	C_10_H_20_O	Fatty, floral-orange odor	0.0030
	Heptanal	C_7_H_14_O	Penetrating fruity, fatty, pungent odor	0.0028
	Dodecalactone	C_12_H_22_O_2_	Fruity, peach-like, pear-like odor	0.0004
	Nonyl acetate	C_11_H_22_O_2_	Mushroom and gardenia aroma and scent	0.0002
Alcohols	2-Ethyl-1-hexanol	CH_3_(CH_2_)_3_CH(CH_2_CH_3_)CH_2_OH	Mild, oily, sweet, slightly floral odor	0.8000
	(Z)-2-Penten-1-ol	C_5_H_10_O	Green diffusive odor	0.7200
	Ethanol	CH_3_CH_2_OH	Pleasant, fragrant, weak, vinous odor	0.6200
	Phenylethyl alcohol	C_6_H_5_CH_2_CH_2_OH	Characteristic rose-like odor	0.5642
	*n*-Butanol	CH_3_(CH_2_)_3_OH	Rancid, sweet, mildly alcoholic odor	0.4800
	1-Hexanol	CH_3_(CH_2_)_4_CH_2_OH	Sweet alcohol, aromatic, pleasant odor	0.0056
	3-Methyl-1-butanol	(CH_3_)_2_CHCH_2_CH_2_OH	Disagreeable odor	0.0040
	2-Methyl-1-butanol	CH_3_CH_2_CH(CH_3_)CH_2_OH	Cooked roasted odor with fruity or alcoholic undertones	0.0040
	1-Octen-3-ol	C_8_H_16_O	Sweet earthy odor	0.0015
Nitrogen Compounds	Methyl-pyrazine	C_5_H_6_N_2_	Nutty, cocoa-like odor	30.0000
	Ethyl-pyrazine	C_6_H_8_N_2_	Musty, nutty, peanut butter odor	4.0000
	2,5-Dimethyl-pyrazine	C_6_H_8_N_2_	Earthy, potato-like odor	1.7500
	2,3-Dimethyl-pyrazine	C_6_H_8_N_2_	Nutty, cocoa-like odor	0.8800
	Trimethylamine	(CH_3_)_3_N	Fishy, amine odor	0.6300
	1-Butanamine	C_4_H_11_N	Fishy, ammonia-like odor	0.5100
	Trimethyl-pyrazine	C_7_H_10_N_2_	Roasted nut, baked potato odor	0.3500
	2,3-Dimethyl-5-ethyl-pyrazine	C_8_H_12_N_2_	Deep roasted cocoa-like aroma	0.2000
	2-Ethyl-6-methyl-pyrazine	C_6_H_8_N_2_	Roasted baked potato odor	0.0400
	2-Ethyl-5-methyl-pyrazine	C_7_H_10_N_2_	Nutty, roasted, grassy odor	0.0160
Sulfur Compounds	Methanethiol	CH_4_S	Powerful, decayed cabbage odor	0.0010
Hydrocarbons	1,2-Dimethylbenzene	C_8_H_10_	Aromatic odor	0.4500
	1,2,4,5-Tetramethyl-benzene	C_10_H_14_	Camphor-like odor	0.0870

## Data Availability

No new data were created or analyzed in this study. Data sharing is not applicable to this article.
